# Self-Subcutaneous Injection of Elemental Mercury: A Report of a Rare Case From Pakistan

**DOI:** 10.7759/cureus.116436

**Published:** 2026-09-17

**Authors:** Rimsha Basharat, Muhammad Shahid, Tooba Waseem, Shahid Rafique, Asma Saleem

**Affiliations:** 1 Department of Anesthesiology and Perioperative Medicine, Al-Khidmat Hospital, Sahiwal, PAK; 2 Department of Anesthesia and Intensive Care Unit, Sahiwal Teaching Hospital, Sahiwal, PAK; 3 Department of Psychiatry, Sahiwal Teaching Hospital, Sahiwal, PAK; 4 Department of Surgery, Sahiwal Teaching Hospital, Sahiwal, PAK; 5 Department of Surgery, Al-Khidmat Hospital, Sahiwal, PAK

**Keywords:** case report, elemental mercury, foreign body granuloma, mercury toxicity, psychiatric evaluation, self-injurious behavior, soft tissue mercury deposition, subcutaneous injection, surgical debridement

## Abstract

Deliberate subcutaneous injection of mercury by a mentally healthy individual is rare. It has been frequently reported in individuals with severe depressive disorders during suicide attempts, as well as among athletes seeking performance enhancement and in certain contexts related to sexual gratification. Here, we present the case of a 28-year-old male patient who deliberately self-injected mercury subcutaneously into his abdomen that led to the formation of a painful induration/abscess in the midline of the abdomen. X-ray of the abdomen showed multiple subcutaneous radio-opaque deposits. A surgical procedure was thereby performed to treat the abscess. However, the patient did not develop the clinical signs of chronic poisoning, suggesting that subcutaneous mercury injection may carry a low risk of systemic toxicity compared with other routes of exposure. The patient denied any psychiatric illness and did not disclose the motive for mercury injection. The patient also has a prior history of hospital admission in a different city for chest wound debridement following mercury injection. The report highlights the timely psychiatric referral and evolution of every patient of subcutaneous mercury injection to avoid repeated hospital presentations and the possibility of systemic toxicity. To our knowledge, this is the first reported case of deliberate subcutaneous elemental mercury injection from Pakistan.

## Introduction

Mercury is a naturally occurring metallic element with an atomic number of 80. It is a unique heavy metal that exists in several forms, including elemental (metallic) mercury, inorganic mercury compounds, and organic mercury compounds. Elemental mercury is a silvery-white, odorless liquid at room temperature and is characterized by its high density and volatility. Mercury has historically been used in thermometers, barometers, electrical equipment, industrial processes, and various medical applications. Exposure to mercury can occur through inhalation, ingestion, and injection [1]. Mercury toxicity is determined by the chemical form of mercury, the magnitude of exposure, and the route of entry into the body [1,2]. A substantial body of literature has documented the toxic effects of mercury on multiple organ systems, including the nervous, digestive, and immune systems, as well as the kidneys, lungs, and skin [1,3]. Diagnosis is typically established through laboratory investigations, including analysis of blood, urine, or hair samples to assess exposure levels [1,2]. Management primarily focuses on prompt identification and elimination of the exposure source, followed by appropriate medical intervention when indicated, including chelation therapy in selected cases [3]. Toxicity resulting from the consumption of methylmercury-contaminated fish in Minamata disease has also been reported [4].

In contrast to the more commonly recognized routes of mercury exposure, intentional subcutaneous injection of elemental mercury is rare [5]. Previously reported cases have described subcutaneous mercury injection in different clinical and behavioral contexts, including deliberate self-harm, suicidal intent, psychiatric or delusional disorders, and attempts to enhance sexual or athletic performance [6,7]. These cases suggest that the motivation and underlying circumstances surrounding mercury injection may vary considerably. Although subcutaneous injection may result predominantly in localized tissue manifestations, systemic absorption cannot be excluded, and systemic complications have been reported in some cases [8,9].

We hereby report a case of intentional recurrent subcutaneous mercury injection by a young man who did not report a clear motive for the injections. The patient presented with localized abdominal and chest wall lesions containing multiple radio-opaque mercury deposits and had a history of a previous similar presentation. Complete recovery was attained following surgical exploration and debridement of the affected subcutaneous sites. No clinically apparent systemic mercury toxicity developed during the observed period; however, blood and urine mercury concentrations could not be obtained, and therefore systemic absorption could not be definitively excluded. The recurrent nature of the injection and the absence of an immediately apparent motive highlight the importance of considering the behavioral and psychiatric context of such cases. Comprehensive psychiatric evaluation and appropriate behavioral follow-up may be important in patients presenting with intentional subcutaneous mercury injection.

## Case presentation

A 28-year-old male patient, a sugar mill technician, presented to our surgery outpatient department with a painful, firm, indurated lesion/abscess in the midline of the abdomen, reportedly containing mercury, which had been present for approximately 1.5 months. The patient was aware that the lesion contained mercury based on his history of a previous surgical procedure for a similar lesion involving the chest region; however, he did not provide information regarding the manner or circumstances of mercury introduction in the present episode. The exact quantity of mercury was also unknown. There was no history of recent trauma or substance abuse (Figure 1). The patient reported a history of a comparable indurated lesion over the right chest wall, which had been treated surgically at a tertiary care hospital approximately 1.5 years previously. Apart from this previous operative procedure, he had no known history of major or chronic medical illnesses. There was no reported history of psychiatric illness or previous psychiatric treatment. He denied any history of substance abuse or recent trauma. He was not taking any regular or relevant medications. Family medical and psychiatric history was unremarkable, with no significant family history of chronic medical or psychiatric disorders. Regarding family and household relationships, the patient reported a strained relationship with his stepmother, while his relationships with other family and household members were described as normal. Socially, the patient primarily focused on his occupational duties and household responsibilities. He reported having a few friends, although none were described as close. He did not report participation in any specific recreational or other structured social activities. His activities of daily living were independent. He worked from approximately 9:00 AM to 5:00 PM and spent the remainder of his time attending to household chores and other necessary responsibilities. The patient was employed as a technician at a sugar mill, where he operated machinery, most of which was digitally controlled. He reported that only one machine at his workplace contained a mercury thermometer, while the other machinery used digital monitoring systems. There was no reported change in his personality or behavior during the preceding 1.5-2 years. The only notable interpersonal change reported was his relatively distant relationship with his stepmother. Regarding the previous episode, only a discharge slip and a chest X-ray from the prior hospitalization, approximately 1.5 years earlier, were available. The discharge documentation contained information regarding the surgical procedure and medications prescribed for a few days after the operation. No records of toxicological investigations or psychiatric assessment were available, and no psychiatric evaluation had been performed during that hospitalization. According to the patient, the nature of the previous chest wall lesion was not known to him at that time, and he was unaware that it contained mercury. In the current episode, however, the patient was certain that the abdominal lesion contained mercury based on his previous experience with the similar chest wall lesion. He did not provide a specific reason or motive for the current or previous episode, nor did he describe the circumstances by which the mercury was introduced.

**Figure 1:**
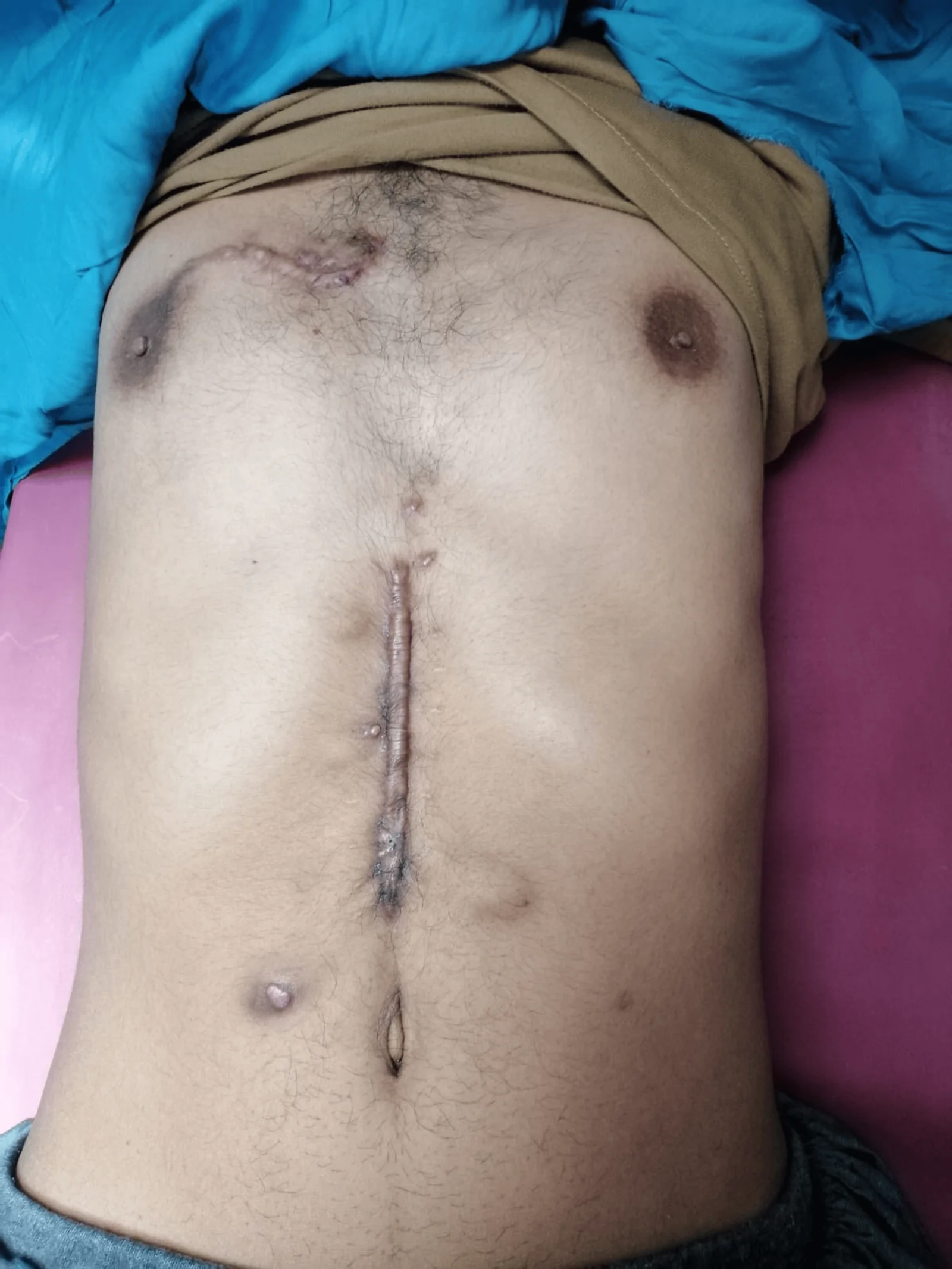
Clinical photograph demonstrating a painful indurated swelling with abscess formation in the midline anterior abdominal wall and a scar mark on the right upper chest wall.

His general physical and systemic examinations were unremarkable, with stable vital signs. On local examination, there was a midline induration/abscess extending from the xiphoid process to two fingers above the umbilicus, measuring 7×2 cm. The induration was tender, firm, and fixed. No local lymphadenopathy was noted. A scar mark of 3×2 cm over the right upper chest wall was also noted (Figure 1). X-ray and ultrasound of the abdomen showed a diffuse area of multiple subcutaneous radio-opaque deposits up to the xiphoid sternum. Chest X-ray demonstrated similar radio-opaque deposits over the right upper chest wall, albeit less extensive than those observed in the abdominal region (Figures 2-4). Routine laboratory investigations, including complete blood count (CBC), liver function tests (LFTs), renal function tests (RFTs), coagulation profile, and viral markers, were performed and were within normal limits. Blood and urine mercury concentrations could not be measured because specialized mercury testing was not available at our institution or at any laboratory in the surrounding area. Therefore, a specific toxicological assessment of systemic mercury exposure could not be performed. The patient was admitted for surgical debridement of the affected subcutaneous tissue and further evaluation and management. Excision of the skin and subcutaneous tissue containing the granulomatous lesion was performed (Figures 5-7). The skin was left open, and the excised tissue was submitted for histopathological examination. The surgical wounds subsequently healed uneventfully. The histopathology report showed acute on chronic inflammation with a few black spherical to oval globules surrounded by a foreign body type of giant-cell reaction (Figure 8).

**Figure 2:**
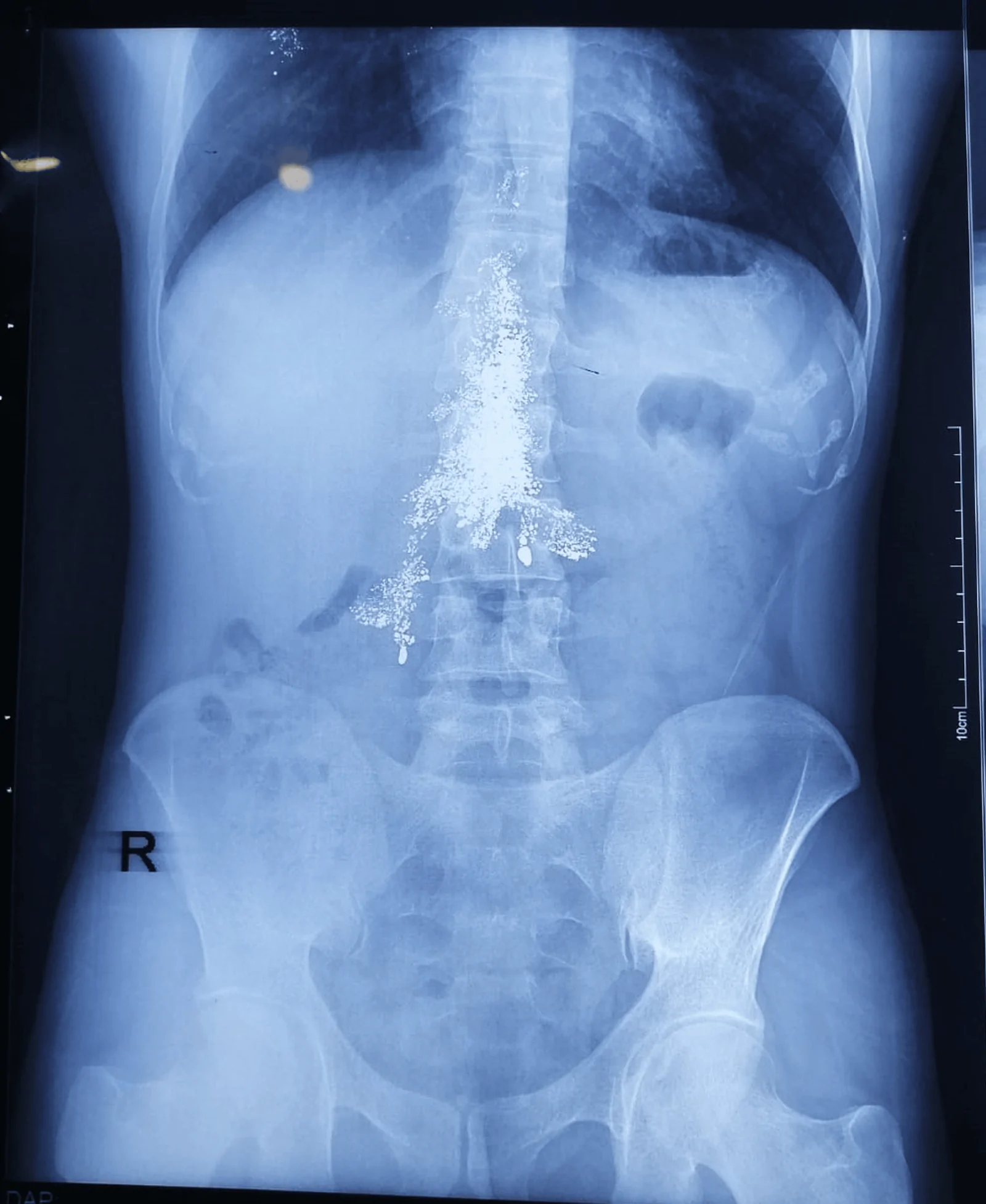
Plain abdominal radiograph showing multiple radio-opaque metallic deposits in the anterior abdominal wall consistent with subcutaneous mercury injection.

**Figure 3:**
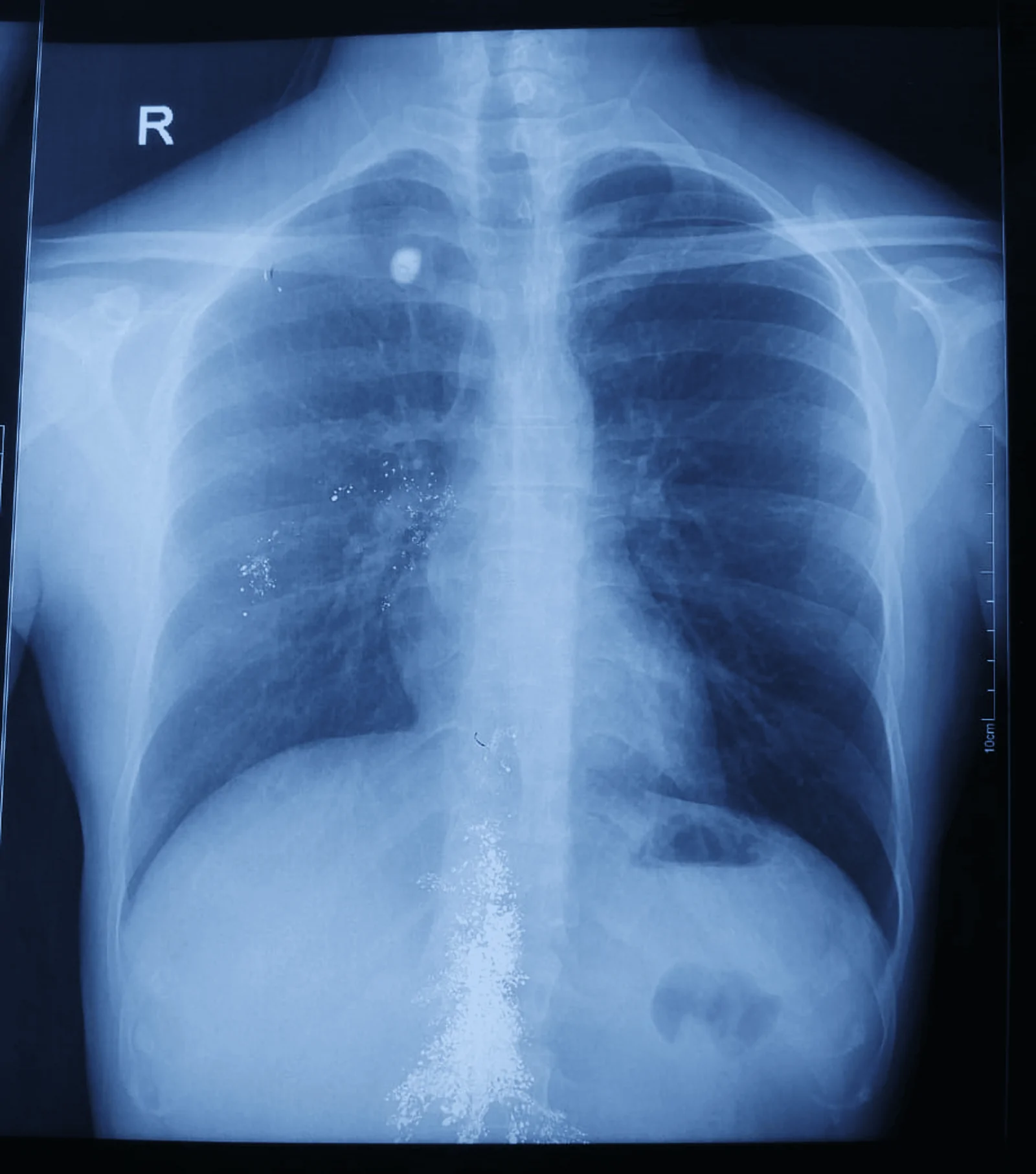
Plain chest radiograph showing multiple but less opaque deposits at the upper right chest wall.

**Figure 4:**
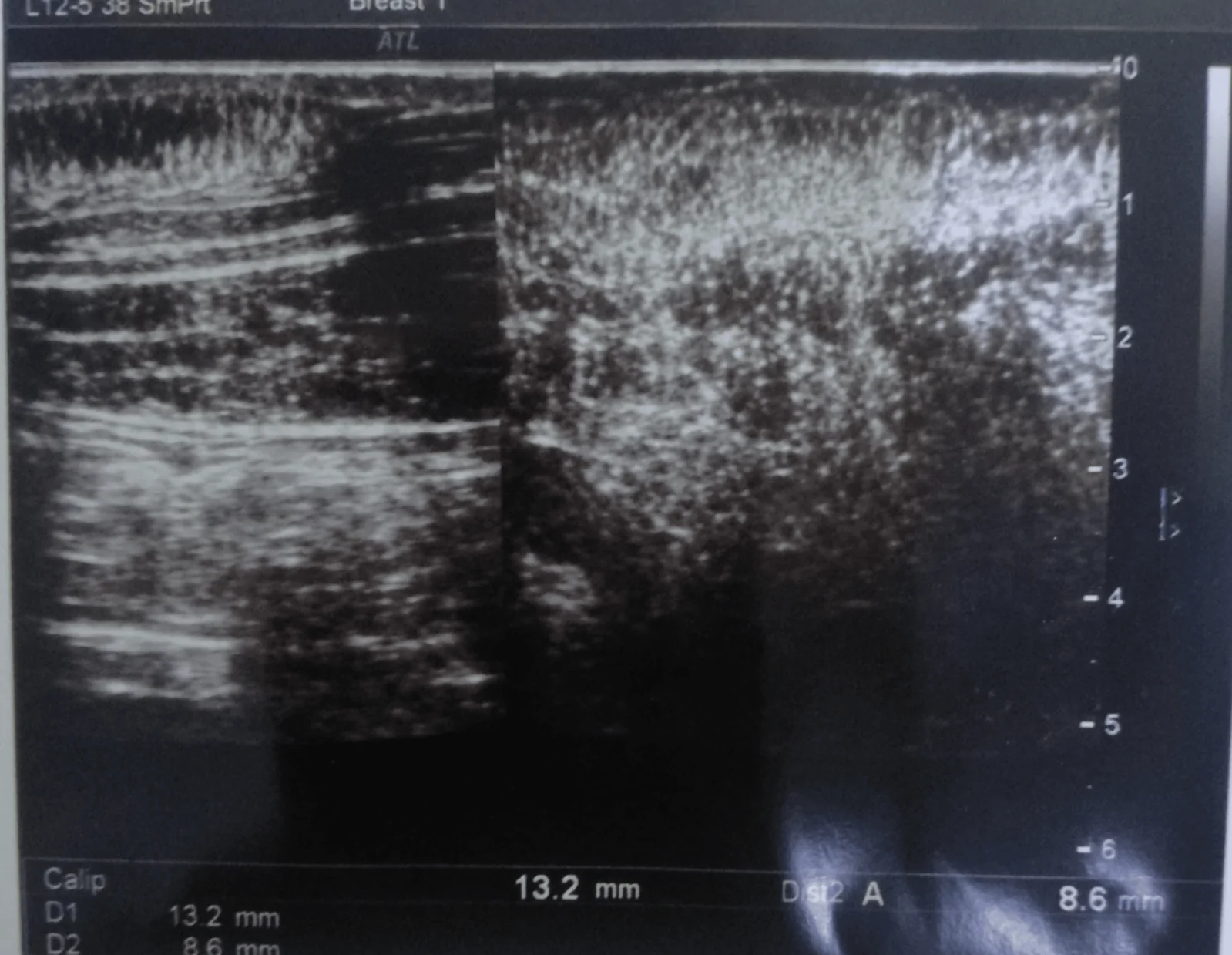
Abdominal ultrasound showing multiple echogenic foci in the anterior abdominal wall.

**Figure 5:**
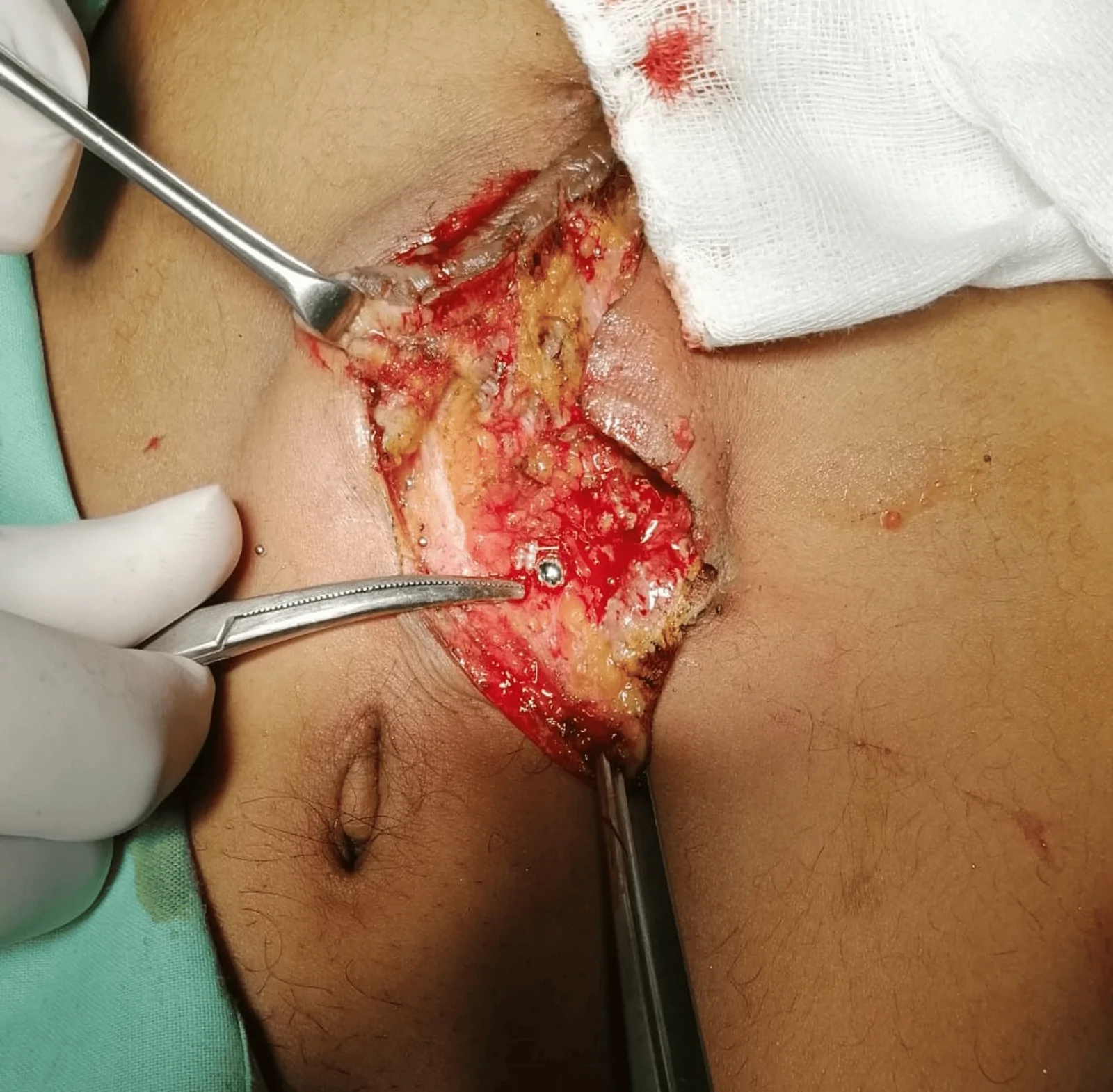
Intra-operative image showing metallic mercury globules within the subcutaneous tissue following surgical debridement.

**Figure 6:**
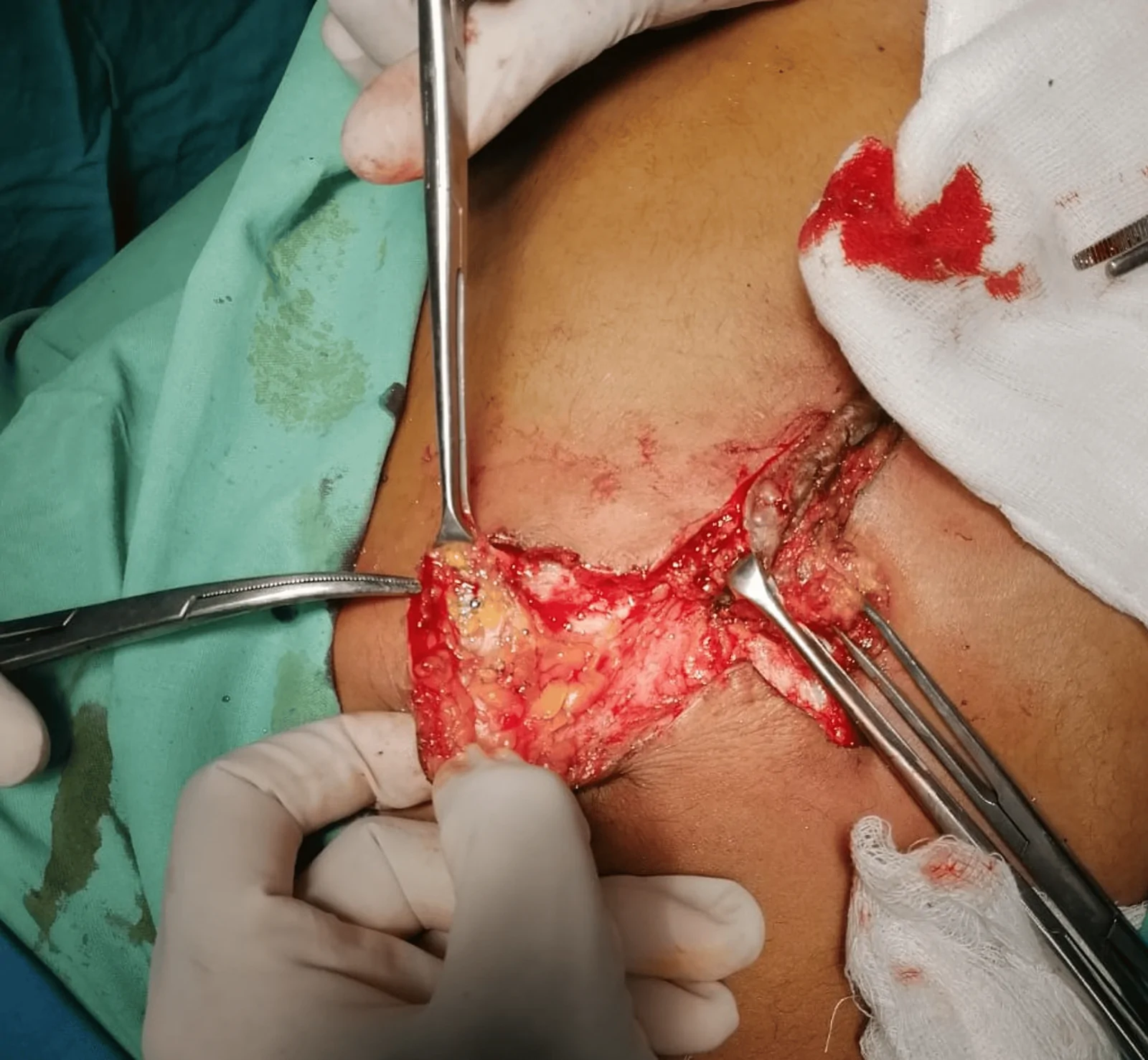
Intra-operative image showing metallic mercury globules within the subcutaneous tissue following surgical debridement.

**Figure 7:**
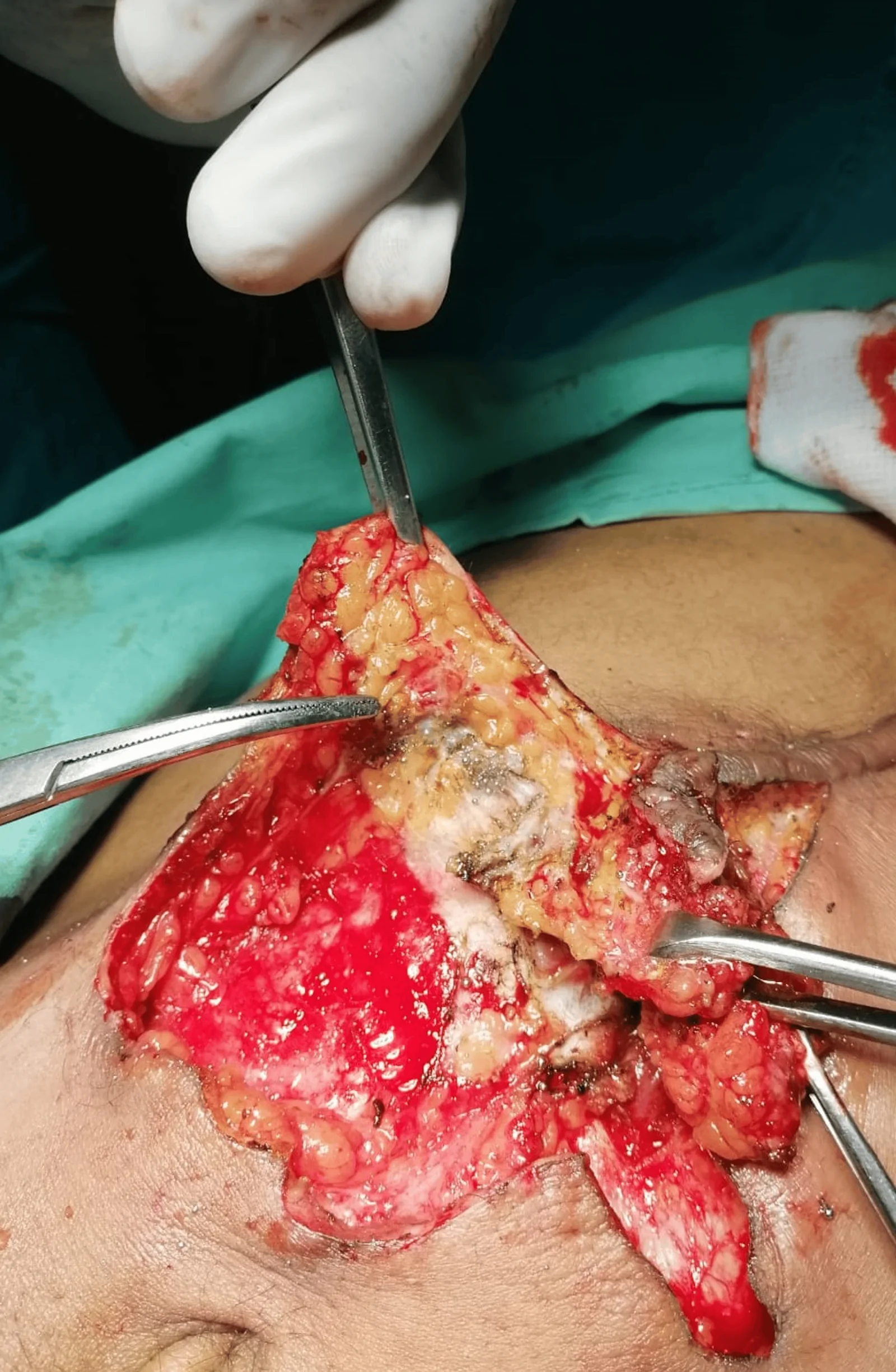
Intra-operative image showing metallic mercury globules within the subcutaneous tissue following surgical debridement.

**Figure 8:**
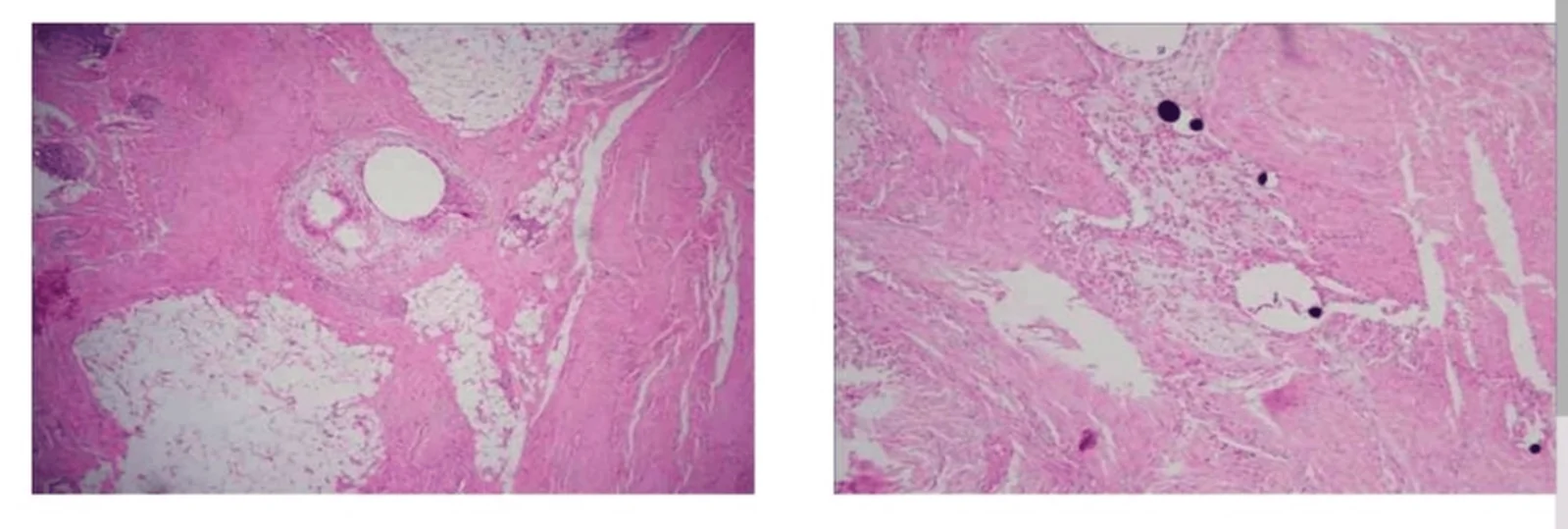
Histopathological image showing black spherical to oval mercury globules surrounded by foreign body giant-cell reaction and chronic inflammatory infiltrate.

Given that this was the patient's second hospital admission for a similar presentation, a formal psychiatric evaluation was undertaken. However, the patient declined to disclose the motive or circumstances underlying the mercury exposure. No evidence of a major psychiatric illness was identified during the assessment. Cognitive function was assessed clinically with respect to orientation, attention, memory, comprehension, and the ability to provide coherent and relevant responses. The patient was alert and appropriately oriented, demonstrated adequate attention and comprehension, and provided relevant and consistent responses, with no clinically apparent cognitive impairment. Depression screening using the Beck Depression Inventory-II (BDI-II) was within normal limits, with a score of 9/63. A formal risk assessment was not conducted, which represents a limitation of this report and limited the systematic assessment of factors potentially contributing to the recurrent exposure. The patient was recommended regular psychiatric and behavioral follow-up, with weekly follow-up for two months followed by monthly follow-up to monitor his clinical status and reduce the risk of recurrence. Social support and continued follow-up were also recommended to address potential behavioral factors associated with the recurrent exposure.

## Discussion

Elemental mercury exposure through self-subcutaneous injection is an uncommon mode of intoxication and has been reported only rarely in the literature [10]. Most documented cases are associated with suicidal intent, severe psychiatric illness, attempts to enhance athletic or sexual performance, or self-injurious behavior [11-13]. In the present case, the patient repeatedly injected mercury into subcutaneous tissues despite the absence of overt psychiatric illness or cognitive impairment, making this presentation particularly unusual. To the best of our knowledge, this is the first reported case from Pakistan.

The toxic effects of mercury depend on its chemical form, route of exposure, quantity, and duration of contact [1]. Elemental mercury is poorly absorbed through intact skin and gastrointestinal mucosa. However, inhalational exposure can lead to severe systemic toxicity due to rapid pulmonary absorption [2]. In contrast, subcutaneous injection usually results in localized inflammatory reactions including abscess formation, granuloma formation, induration, cellulitis, and foreign body giant-cell response surrounding mercury globules.

Radiological imaging plays an important role in diagnosis because elemental mercury appears as dense radio-opaque deposits on X-ray imaging [14]. Similar radiographic findings were observed in our patient, with multiple scattered subcutaneous radio-opacities in the abdominal wall and previous chest wall deposits. Imaging not only aids in confirming the diagnosis but also assists in surgical planning for the removal of deposited mercury [14].

Although localized mercury deposition generally carries a low risk of systemic toxicity, systemic absorption may occasionally occur through lymphatic or vascular channels, potentially leading to nephrotoxicity, neurotoxicity, or pulmonary complications [15,16]. Clinical manifestations of systemic mercury poisoning include tremors, insomnia, memory impairment, peripheral neuropathy, irritability, and renal dysfunction [1]. Fortunately, our patient did not exhibit any clinical evidence of systemic involvement during hospitalization or follow-up. Similar observations have been documented in previous reports where localized injections remained confined to soft tissues without significant systemic absorption [6,10].

Management of subcutaneous mercury injections remains controversial because no standardized guidelines exist [17]. However, most authors recommend prompt surgical excision and debridement of accessible mercury-containing tissue to minimize local tissue damage and reduce the possibility of absorption [1,13]. In the present case, complete surgical exploration and debridement resulted in satisfactory wound healing without complications. Chelation therapy is generally reserved for patients demonstrating elevated mercury levels or systemic toxicity [1,3]. A notable limitation of this case was the inability to measure blood and urine mercury concentrations because of the lack of local laboratory facilities. Consequently, objective assessment of systemic mercury absorption could not be performed. However, the patient remained clinically asymptomatic, with no evidence of systemic toxicity during follow-up, reducing the immediate indication for chelation therapy.

An important aspect highlighted by this case is the role of psychiatric evaluation in patients presenting with intentional or recurrent self-injection injuries. Previous reports have described mercury self-injection in association with psychiatric disorders, self-harm behavior, and other behavioral disturbances [10,12]. Although our patient denied psychiatric symptoms and demonstrated normal cognitive functioning, the recurrent nature of his presentation warranted psychiatric evaluation and continued follow-up. Psychiatric assessment is important in such cases to identify underlying psychopathology, assess potential self-harm risk, and explore behavioral factors that may contribute to recurrent exposure. In this patient, psychiatric assessment was undertaken to explore possible factors contributing to the recurrent exposure and to guide appropriate follow-up and supportive management.

This case emphasizes that subcutaneous mercury injection may present primarily as a localized surgical problem without overt systemic manifestations. Clinicians should maintain a high index of suspicion when encountering unexplained indurated lesions with radio-opaque deposits on imaging. A multidisciplinary approach involving surgeons, toxicologists, radiologists, and psychiatrists is essential for optimal management and the prevention of recurrence.

## Conclusions

Self-subcutaneous injection of elemental mercury is a rare form of self-inflicted injury that usually presents with localized inflammatory reactions rather than overt systemic toxicity. Early recognition through clinical suspicion and radiological imaging is important for timely surgical management and prevention of further complications. Although systemic absorption is uncommon in subcutaneous exposure, repeated injections may increase the risk of chronic mercury toxicity. This case also highlights the importance of considering psychiatric assessment and long-term follow-up in patients presenting with deliberate, recurrent, or unexplained subcutaneous mercury exposure, particularly when the motivation for exposure is unclear. Psychiatric assessment may help identify underlying psychopathology, self-harm risk, or behavioral factors and guide appropriate management and follow-up. To the best of our knowledge, based on a literature search of PubMed, Google Scholar, and other relevant published medical literature using combinations of the terms "subcutaneous mercury injection", "mercury injection", "elemental mercury", and "Pakistan", no previously reported case of subcutaneous mercury injection from Pakistan was identified. Therefore, we consider this to be the first reported case from Pakistan.
